# Méga-urètre congénital compliqué de calculs: à propos d'un cas

**DOI:** 10.11604/pamj.2019.33.170.19229

**Published:** 2019-07-04

**Authors:** Yacoub Mohamed Sghair, Med Elmoktar Mballa, Sidi Mohamed Naji, Ahmed Kane

**Affiliations:** 1 Service de Chirurgie Pédiatrique, Centre Hospitalier Mère et Enfant, Nouakchott, Mauritanie; 2 Service d'Urologie, Hôpital d'Amitié, Nouakchott, Mauritanie

**Keywords:** Méga-urètre congénital, calcul, urétroplastie, Congenital mega urethra, stone, urethroplasty

## Abstract

Le méga-urètre congénital est une malformation rare à l'origine d'un désordre mictionnel avec parfois un retentissement sur le haut appareil urinaire. Malgré l'association fréquence avec d'autres malformations urinaires et extra urinaires, chez l'enfant, la complication par des calculs n'a été rapportée qu'une seule fois dans la littérature. Nous rapportons un cas de méga-urètre congénital compliqué de calculs de stase chez un enfant de 3 ans pris en charge avec succès au Service de Chirurgie Pédiatrique du Centre Hospitalier Mère et Enfant de Nouakchott. A travers cette observation, nous revenons sur les aspects épidémiologiques, cliniques et thérapeutiques de cette association entre méga-urètre congénital et calculs urinaires.

## Abstract (en)

Congenital mega-urethra is a rare malformation causing micturition disturbance sometimes associated with upper urinary tract impairment. Despite the frequent association with other urinary and non-urinary abnormalities in children, stone-related complication has been reported only once in the literature. We report a case of congenital mega urethra complicated by stasis and stone formation in a 3 year old child successfully treated in the Department of Paediatric Surgery at the Mother-Child Hospital in Nouakchott. This study highlights the epidemiological clinical and therapeutic features of this association between congenital mega-urethra and urinary stones.

## Introduction

Le méga-urètre congénital (MUC) est une dilatation de la partie terminale d'urètre pénien et de l'urètre glandulaire associée à un méat urétral en place. Cette dilatation est due à l´absence de développement des tissus érectiles péniens dont l'étiologie demeure inconnue [1]. En fonction de la forme de la dilatation urétrale, on distingue deux types de MUC, scaphoïde et fusiforme. Ces deux formes ont un pronostic différent [2]. Il s'agit d'une pathologie rare qui est à l'origine d'un désordre mictionnel avec parfois un retentissement sur le haut appareil urinaire. La formation des calculs au sein de la dilatation est exceptionnelle. Elle est due à la stase permanente des urines. Nous rapportons le cas d'un garçon de 3 ans atteint de méga-urètre scaphoïde qui a développé deux calculs secondaires et pris en charge au Service Chirurgie Pédiatrique du Centre Hospitalier Mère et Enfant de Nouakchott.

## Patient et observation

**Figure 1:**
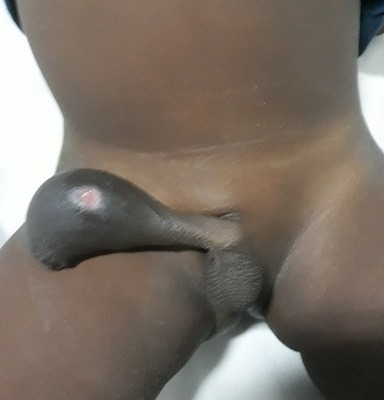
Dilatation scaphoïde de de la verge

**Figure 2:**
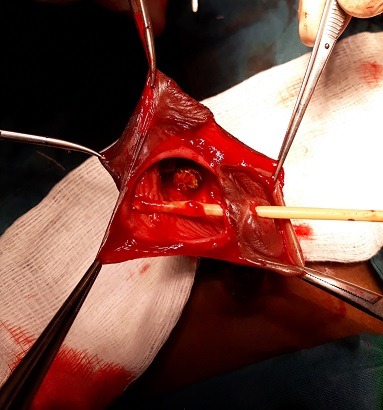
Dissection de l'urètre avec la présence de calculs

**Figure 3:**
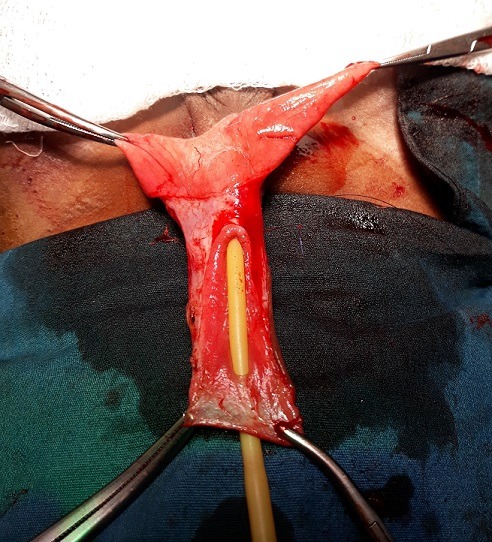
Urètre après la résection de l'excès v

**Figure 4:**
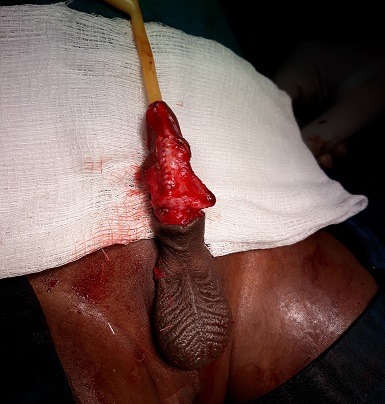
Uretroplastie sur sonde Foley

**Figure 5:**
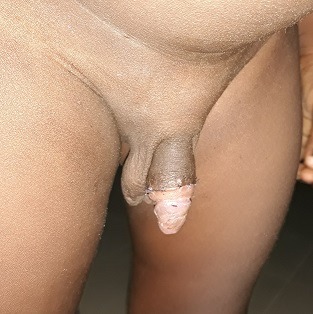
Aspect définitif de la verge

Il s'agit d'un garçon âgé de 3 ans qui a consulté pour une tuméfaction ulcérée sacciforme de la partie distale de la verge (Figure 1) associée à une dysurie et un jet urinaire faible. L'examen clinique a retrouvé une formation kystique pénienne molle et indolore. Les testicules étaient en place. Il n'y avait pas de globe vésical. L'échographie abdominale était normale tandis que l'échographie pénienne a mis en évidence une dilatation de l'urètre distal avec la présence de deux calculs péniens. La fonction rénale était normale. Le geste opératoire a consisté en une incision circonférentielle à la jonction tiers moyen et tiers inférieur de la verge associée à une mise en place d'une sonde urinaire N° 8. L'ouverture médiane de la partie dilatée a permis de constater la présence de deux calculs de 10 mm et 5 mm respectivement (Figure 2). Nous avons procédé à l'exérèse de la partie excédentaire de l'urètre (Figure 3), associée à une uretroplastie sur la sonde urinaire par un surjet en mono-filament résorbable 5/0 (Figure 4). Enfin nous avons réalisé une circoncision réglée. Les suites opératoires ont été simples permettant l'ablation de la sonde au septième jour post opératoire avec une miction normale et un bon jet urinaire (Figure 5).

## Discussion

Le méga-urètre congénital est une pathologie très rare [3]. Il réalise une obstruction fonctionnelle entrainant la stase des urines dans le segment dilaté. Sur le plan embryologique, il s'agit d'un défaut de développement des tissus érectiles d'origine mésenchymateuse au sein desquels chemine l'urètre [4]. Stephens a décrit deux formes anatomiques selon l'importance du déficit des formations érectiles [2]: la forme sacciforme avec des corps caverneux normaux et la forme fusiforme, la plus grave qui est associée à une agénésie des corps caverneux responsable d'une ectasie de l'urètre sur toute sa circonférence. Notre patient présentait une forme sacciforme avec agénésie partielle mais isolée du corps spongieux. Sur le plan clinique, Il s'agit souvent d'une déformation monstrueuse de la verge avec déviation qui attire l'attention dès la naissance. Parfois, le diagnostic est fait tardivement suite à des complications telles qu'une rétention urinaire, une dysurie ou une infection urinaire récidivante voire une fistule post circoncision. La survenue de calcul est exceptionnelle que ça soit au niveau urétral ou au niveau du haut appareil urinaire. Kolte *et al*. [5] ont rapporté cette association MUC et calcul urétrale chez un enfant de 8 ans. Ces calculs sont en rapport avec la stase. Dans notre cas le diagnostic a été fait devant la dysurie, malgré que les parents fussent conscients que la verge de leur enfant n'était pas normale. En plus de la fonction rénale, d'autres explorations radiologiques sont nécessaires à savoir l'échographie abdominale et l'urétro-cystographie rétrograde (UCR) à la recherche des malformations associées et pour évaluer le retentissent su le haut appareil urinaire [6-8]. La chirurgie de correction doit être entamée en période néonatale pour lever l'obstruction fonctionnelle urétrale [9]. Il s'agit d'une uretroplastie modelante selon la technique de Nesbitt [1] et Locke [10] qui consiste à une dissection avec réduction du calibre urétral, une résection de l'excès des tissus cutanés et préputiaux suivie par une uretroplastie sur une sonde. Rarement une dérivation urinaire est nécessaire pour pallier à un retentissement majeur sur le haut appareil. Notre patient a été opéré selon la technique de Nesbitt à l´âge de 3 ans et les suites opératoires étaient simples avec un bon aspect cosmétique et un bon jet urinaire.

## Conclusion

Le méga-urètre congénital est une pathologie rare qui mérite d'être étudiée et connue. Son diagnostic est clinique. Le traitement est chirurgical et doit être mis en route avant l'installation de distorsion urétrale ou d'un retentissement sur le haut appareil. La recherche des malformations mésodermiques parfois associées doit être systématique.

## Conflits d’intérêts

Les auteurs ne déclarent aucun conflit d’intérêts.
